# Dairy Consumption: Does It Make an Impact on Self-Reported Disease Activity of Inflammatory Arthritis?

**DOI:** 10.7759/cureus.15010

**Published:** 2021-05-13

**Authors:** Steve S Kong, Matthew Robinson, Tyler Hosterman, Neha Bhanusali

**Affiliations:** 1 Internal Medicine, Highland Hospital, Oakland, USA; 2 Biostatistics, University of Central Florida College of Medicine, Orlando, USA; 3 Internal Medicine, University of Miami Miller School of Medicine, Miami, USA; 4 Rheumatology, University of Central Florida College of Medicine, Orlando, USA

**Keywords:** inflammatory arthritis, diet therapy, joint diseases, psoriatic arthritis, rheumatoid arthriitis

## Abstract

Background

As researchers and the public become more cognizant of the impacts of diet and nutrition on health, continued research is needed to provide evidence to support dietary claims. At present, there exists mixed reporting on the effects of dairy consumption and disease activity of inflammatory arthritis (IA).

Objective

This study attempts to advance current research on the relationship between dairy consumption and self-reported disease activity in patients with IA and to investigate whether dietary modifications can be helpful as a conservative, cost-effective, and accessible supplement to established treatments.

Methods

Participants completed a modified diet history questionnaire (DHQ), which assessed dairy consumption over the past year, and a Routine Assessment of Patient Index Data 3 (RAPID3) questionnaire, which assessed a participants’ self-reported inflammatory disease activity. DHQ and RAPID3 were analyzed using a Pearson product-moment partial correlation to assess variables’ relationships. Participants completed the questionnaire in the setting of a rheumatology clinic. Two hundred and four participants were recruited for this study. All of the participants were at least 18 years of age, capable of giving informed consent, and were formally diagnosed with either rheumatoid arthritis or psoriatic arthritis by a board-certified rheumatologist.

Results

The results from the questionnaires found that dairy consumption does not contribute to self-reported IA disease activity. While 11 of the 16 DHQ variables maintained a positive correlation with the overall RAPID3 scores, none of these possessed statistical significance. Only when controlling for age and sex did the study find two statistically significant variable correlations between the quantity of milk consumed as a beverage (r=0.147, n=193, p=0.043) and milk added to cereal (r=0.170, n=189, p=0.019) with the RAPID3 scores.

Conclusion

In summation, the study found no notable correlation between dairy consumption and patients’ self-reported IA disease activity.

## Introduction

Inflammatory arthritis (IA) is a group of chronic autoimmune diseases that affects the joints and the surrounding tissues. IA affects 2%-3% of the general population and is associated with a wide range of comorbidities and a decrease in the quality of life [1,2]. While there are many types of IA, the most common types in adults include rheumatoid arthritis (RA) and psoriatic arthritis (PsA) [3]. The mainstay of management of IA has focused on disease-modifying anti-rheumatic drugs (DMARDs) and non-steroidal anti-inflammatory drugs (NSAIDs) [4,5]. Over the last 20 years, great strides have been made in the pharmacologic management of IA with the introduction of biological DMARDs and targeted synthetic DMARDs [6,7]. Despite this, access to DMARDs is often limited due to their high cost [8,9]. Additionally, not all patients respond to DMARDs with favorable outcomes and DMARDs can have serious side effects, such as the increased risk of cardiovascular events, infections, and malignancies [10,11]. Thus, a need still exists for additional therapeutic options in the management of IA.

Diet plays an important role in the disease activity of IA [12,13]. Various diet regimens are believed to have anti-inflammatory effects and patients with IA often make modifications to their diet in hopes to lessen the disease activity. Popular dietary regimens include vegan, Mediterranean, and elimination diets. Some diet regimens, such as the Mediterranean diet, have shown promising outcomes in patients with IA, with a statistically significant reduction in pain scores and morning stiffness [14]. However, other diets, such as elimination diets and vegan diets, have demonstrated mixed results [15,16]. A recent study by Winkvist found that disease activity of RA, as measured by Disease Activity Score in 28 joints - erythrocyte sedimentation rate (DAS28-ESR), was lower after 10 weeks of an anti-inflammatory diet (composed of fish, whole-grain cereals, vegetables, and low-fat dairy) than before the intervention [17].

Of particular interest for this study is the effect of dairy on IA. Studies into the effect of dairy and inflammation have been mixed and often contradictory [18,19]. Dairy has been hypothesized to have pro-inflammatory effects and contribute to an increase in inflammatory biomarkers. This belief can be found throughout popular media and scientific journals [20]. Due to these beliefs, many patients often go to extreme lengths to minimize their dairy consumption [21]. However, recent data have suggested that dairy does not actually lead to an increase in circulatory inflammatory biomarkers [21,22]. In fact, some studies have demonstrated how dairy has either a neutral or a protective effect against inflammatory processes [18,23]. At present, there exists no definitive relationship between dairy and inflammation, nor clear guidelines on the suggested dietary dairy intake for patients with IA. As a result, patients are often left without a clear answer on the recommendations or effects of dairy consumption on IA. This study investigated the role of dairy intake using a modified diet history questionnaire (DHQ) and compared it to the self-reported disease activity of patients with RA and PsA using the Routine Assessment of Patient Index Data 3 (RAPID3) questionnaire.

## Materials and methods

All protocol for the study was reviewed and approved by the Institutional Review Board at the authors’ institution in compliance with the Helsinki Declaration and in accordance with the ethical standards on human experimentation. Written consents of human subjects were obtained from all participants prior to the initiation of the study. Participants were recruited from an ambulatory setting rheumatology clinic, over the course of 10 months. During the participants’ regularly scheduled visits, they were informed about the study and asked if they were interested in participating. Participants were informed that their decision to participate in the study would not impact the level of care they received. However, if they choose to participate, they would be asked to complete two questionnaires-a modified DHQ and a RAPID3 questionnaire (Appendices I, II). The former is an assessment on the participant’s dairy intake frequency and quantity over the past 12 months, while the latter is a measurement tool which assessed patients’ self-reported disease activity of their IA. The RAPID3 questionnaire is a tool that is commonly used in the clinical setting to measure self-reported disease activity. Participants were informed that the two questionnaires should take 10-15 minutes to complete.

Only patients who had been formally diagnosed with either rheumatoid arthritis or psoriatic arthritis by a board-certified rheumatologist were asked to participate in the study. Eligible adult patients, over 18 years of age, capable of giving informed consent were entered into the study and completed the modified DHQ and RAPID3 questionnaires. Patients from vulnerable populations, including patients who are pregnant or prisoners, were not included in the study. Additionally, due to limitations of the questionnaires, only patients who were able to read English were included in the study. 

The primary objective of this study was to evaluate if there was a relationship between dairy consumption and the self-reported disease activity of IA. To this end, the National Institutes of Health (NIH) DHQ was modified to only include questions that were relevant to dairy products. In total, there were 16 questions included in the modified DHQ that asked about both the frequency and the quantity of consumption of various dairy products such as milk, yogurt, cheese, ice cream, and creamers (Appendix I). In order to measure the self-reported disease activity of IA, the routine assessment of patient index data 3 (RAPID3) questionnaire was given to participants (Appendix II). The endpoint of the study was when 200 participants had completed both the modified DHQ and RAPID3 questionnaires.

The NIH DHQ contained questions regarding the frequency of consumption of dairy products in various formats ranging from number of times per year to number of times per week and days. In order to make analysis more consistent, all questions regarding frequencies were converted to the total number of times per year. Likewise, NIH DHQ contained questions assessing the quantity of dairy products consumed per consumption in various formats ranging from cups, tablespoons, and ounces. All questions regarding the quantity were converted to quantity in fluid ounces.

The RAPID3 questionnaire was composed of three categories that quantified 1) self-reported level of difficulty experienced while performing various activities of daily living, 2) self-reported pain experienced during daily activities, and 3) self-reported well-being of the patient. These three categories were scored from 0 to 10 points in each category, for a total of up to 30 points. RAPID3 scores ≤ 3 were considered to be in remission, scores 3-6 were of low severity, scores 6-12 were of moderate severity, and scores >12 were of high severity.

All the data from both questionnaires were collected and analyzed using IBM Statistical Package for the Social Sciences (SPSS) version 24.0 (SPSS Inc., Chicago, IL, USA).

## Results

**Figure 1:**
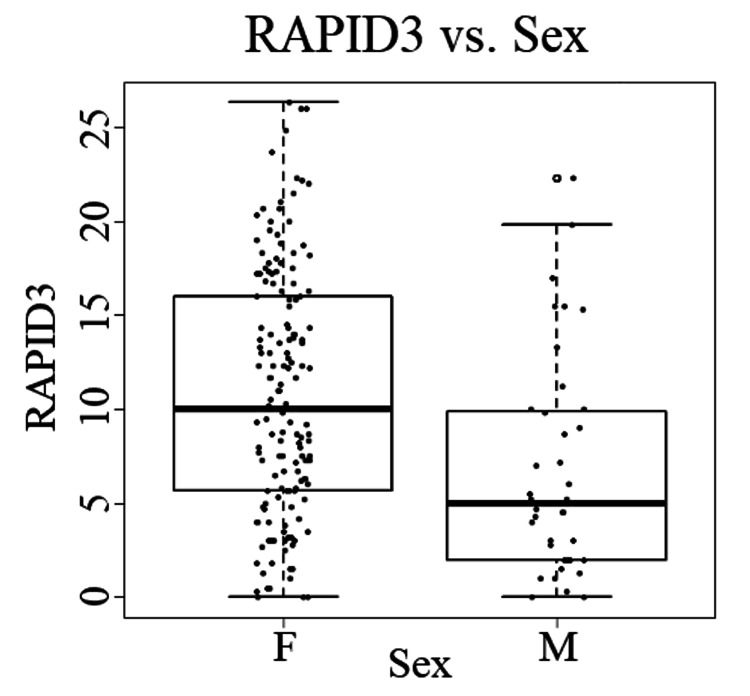
RAPID3 self-reported disease activity vs. sex Females had a statistically significant higher average self-reported overall disease activity of IA (10.8459 ±6.46494) as measured by the RAPID3 questionnaire compared to males (6.7282 ± 5.7893). RAPID3 - Routine Assessment of Patient Index Data 3 IA - inflammatory arthritis

In total, 204 participants completed both the modified DHQ and RAPID3 questionnaires. The average age of the participants were 58.6 years of age, with a median of 58 years of age. There were 157 females, 39 males, and 8 patients who preferred not to answer. The average RAPID3 score was 9.9 with a median score of 8.9. There were 38 participants who were in remission, 33 in low severity, 55 in moderate severity, and 78 in high severity. Male participants had statically significantly lower RAPID3 scores (6.7282 ± 5.7893) compared to female participants (10.8459 ±6.46494), t(194) = -3.631, p = <0.001 (Figure 1).

A Pearson product-moment partial correlation was run to determine the relationship between each of the 16 questions of the modified DHQ and the overall RAPID3 score. There was a positive correlation in 11 of the 16 dairy intake variables of the modified DHQ and the overall RAPID3 scores. However, none of the correlations was statistically significant.

**Figure 2:**
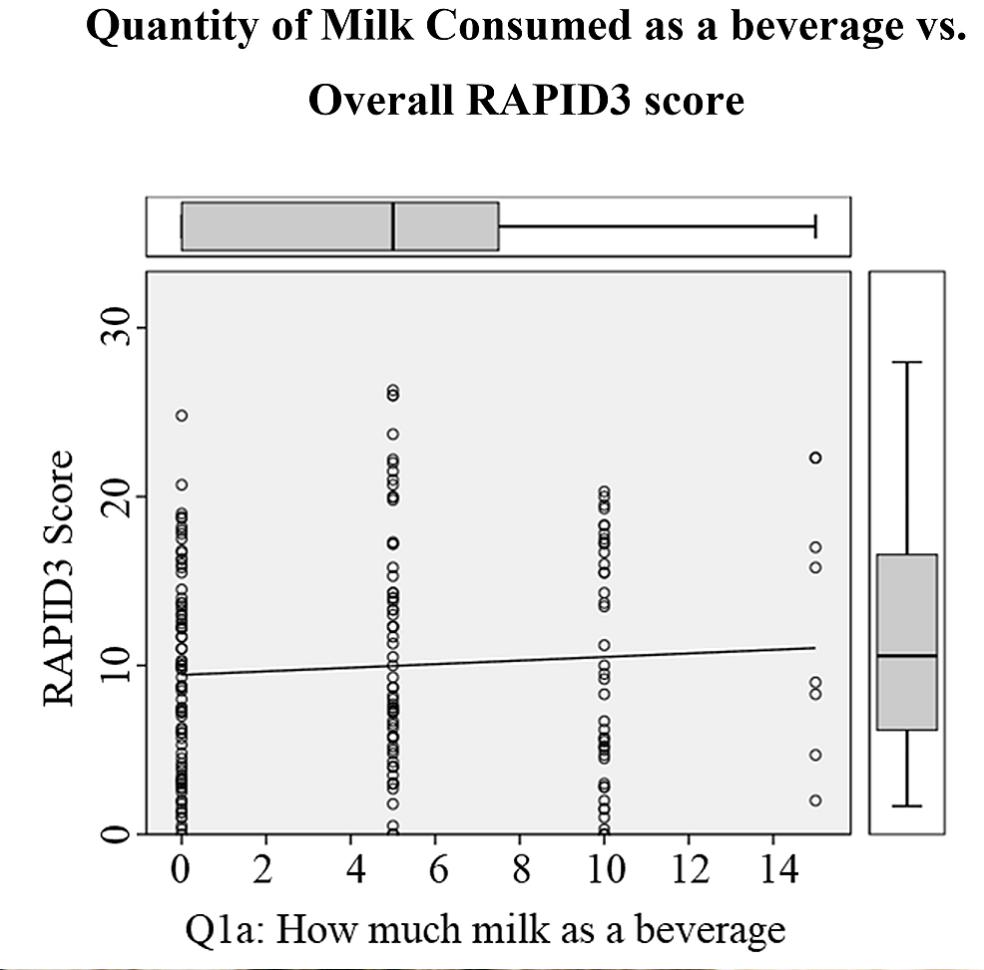
Quantity of milk consumed as a beverage vs. overall RAPID3 score A statistically significant positive correlation was found between the quantity of milk consumed as a beverage and the overall self-reported disease activity measured by the RAPID3 score. The average drinks consumed was (4.35 ± 4.12 fl oz/year) and the overall RAPID3 score (10.04 ± 6.57) whilst controlling for age (58.5 ± 14.6 years) and sex (77% female). This statistically significant positive correlation was only observed after controlling for age and sex. RAPID3 - Routine Assessment of Patient Index Data 3

Next, the Pearson product-moment partial correlation was run comparing the individual variables of the modified DHQ to the overall RAPID3 score whilst controlling for age and sex. There was a positive partial correlation between the quantity of milk consumed as a beverage (4.35 ± 4.12 fl oz/year) and the overall RAPID3 score (10.04 ± 6.57) whilst controlling for age (58.5 ± 14.6 years) and sex (77% female), which was statistically significant, r(189) = 0.147, N = 193, p = .043 (Figure 2). No statistically significant was found with zero-order correlations between the quantity of milk consumed as a beverage and the overall RAPID3 score (r(203)=0.071, n=204, p=0.312) when not controlled for age and sex. Additionally, there was another positive partial correlation identified between the quantity of milk added to cereal and the overall RAPID3 score whilst controlling for age and sex r=0.170 (p=0.019, df=189), which decreased to r=0.160 (p=0.025, df=193) when only controlled for sex. All other correlations between the individual variables of the modified DHQ and the overall RAPID3 scores were non-significant even whilst controlling for age and sex.

Lastly, in order to analyze the individual variables of DHQ further, the Pearson product-moment correlation was run between the individual variables of the modified DHQ and the individual variables of the RAPID3 questionnaire (Table 1). Positive correlations were found between individual variables from the RAPID3 questionnaire and the following questions from the DHQ- quantity of milk consumed as a beverage (Q1a), the quantity of milk added to cereal (Q2d), the quantity of ice cream consumed (Q7a), frequency of milk added to the coffee (Q10), and quantity of milk added to the coffee (Q10a). Additionally, positive correlations were identified between the individual variables from the RAPID3 questionnaire and the age of the participants.

**Table 1:** Pearson correlation value Pearson correlational relationship between individual questions of the diet history questionnaire and the RAPID3 questionnaire. Statistically significant correlations are highlighted in gray. In total, there were 10 statistically significant Pearson correlations. All of the statistically significant correlations were positive correlations, suggesting that increased intake in frequency or quantity in dairy products led to increased self-reported disease activity of IA. (For a complete list of the individual questions on both DHQ and RAPID3 questionnaires please refer to Appendices I, II, respectively.)

Pearson Correlation Value Table															
	RAPID3 Questionnaire														
Diet History Questionnaire		1a.	1b.	1c.	1d.	1e.	1f.	1g.	1h.	1i.	1j.	2	3	RAPID 3 Score	
	Age	0.074	0.005	0.137	0.101	0.075	0.089	.158^*^	.258^**^	.209^*^	.202^*^	0.111	0.076	0.126	
	Q1.	0.079	0.071	0.095	0.015	0.05	0.08	0.05	0.126	0.052	0.086	-0.004	0.001	0.022	
	Q1a.	0.072	0.073	0.115	-0.076	0.116	.158^*^	0.088	0.113	0.08	0.106	0.056	0.051	0.071	
	Q2.	0.068	0.07	0.053	-0.004	0.084	0.04	0.106	0.036	-0.037	-0.026	0.049	0.03	0.044	
	Q2d.	0.127	0.06	0.023	-0.062	0.131	0.054	.166^*^	0.054	0.045	0.11	0.072	0.091	0.094	
	Q3.	-0.028	0.017	-0.029	-0.058	-0.083	0.015	0.049	-0.082	-0.085	0.01	0.08	-0.003	0.017	
	Q3a.	-0.085	0.029	0.062	0.039	0.016	0.092	0.01	-0.011	-0.03	-0.047	0.043	-0.028	0.002	
	Q4.	-0.085	-0.091	-0.023	0.016	0.039	-0.008	-0.012	0.032	0.013	-0.006	-0.056	-0.084	-0.08	
	Q4a.	-0.005	-0.043	0.03	-0.093	-0.015	0.011	-0.021	0.091	-0.021	-0.008	-0.02	0.01	-0.01	
	Q5.	-0.004	0.032	0.078	-0.064	-0.014	-0.024	-0.01	-0.065	0.067	0.064	-0.055	-0.027	-0.028	
	Q5a.	0.017	0.056	0.115	0.058	0.1	0.009	-0.014	-0.016	0.002	0.055	-0.017	0.082	0.038	
	Q7.	0.112	0.079	0.109	0.126	0.049	0.104	0.119	0.038	0.086	0.054	0.047	0.08	0.084	
	Q7a.	0.057	0.104	0.082	0.011	.156^*^	0.07	-0.017	-0.034	0.018	0.032	0.058	0.083	0.072	
	Q9.	0.006	-0.097	0.1	-0.114	-0.04	-0.098	0.024	-0.078	-0.033	-0.074	-0.037	-0.037	-0.052	
	Q9a.	0.065	-0.118	0.011	-0.087	0.005	-0.077	0.02	0.001	0.045	0.061	0.006	-0.055	-0.021	
	Q10.	0.128	0.127	.150^*^	0.021	0.115	0.093	0.102	0.122	-0.024	0.075	.164^*^	0.058	0.116	
	Q10a.	0.074	.176^*^	0.094	0.05	0.066	0.114	0.027	.147^*^	0.063	0.131	0.128	0.077	0.12	

## Discussion

The purpose of this study was to determine if there was a correlational relationship between dairy consumption and self-reported disease activity of IA, specifically RA and PsA. At the conclusion of the study, no statistically significant relationship between the frequency and quantity of consumption of various dairy products and the self-reported disease activity, measured by the overall RAPID3 score, were identified. This finding is in line with recent studies that have suggested that dairy products do not make a clinically significant difference in the disease activity of IA [24].

One potential reason that variations in dairy consumption did not make a difference in the overall disease activity of IA may be the fact that most of the patients in this study were already on pharmacologic therapy. Pharmacologic agents such as DMARDs, biologic agents, and NSAIDs are the first line therapeutic options for RA and PsA [25,26]. Patients often experience favorable outcomes with these pharmacologic agents; and thus, other factors such as variations in dairy consumption may not have played as critical of a role in the self-reported disease activity. Variations in dairy consumption presumably have a lesser impact on the disease activity compared to the impact of directed pharmacologic therapy. Despite this, it is important to note that not all patients experience favorable outcomes with pharmacologic agents. Additionally, for some, pharmacologic agents may not be accessible. In these patients, even minor factors such as variations in diet may have a greater impact on their disease activity.

Another potential reason for the failure to reject the null hypothesis may be that the self-reported disease activity of IA had confounding variables that masked the effects of dairy consumption. In particular, the sex and age of the participants appeared to have an impact on the overall RAPID3 score. Support for this theory was demonstrated when statistically significant relationships were identified with the Pearson product-moment partial correlation whilst controlling for age and sex. Statistically significant relationships between the quantity of milk consumed as a beverage and the overall RAPID3 score and the quantity of milk added to cereal and the overall RAPID3 score were identified, which were not found with zero-order correlations without controlling for age and sex. Furthermore, the positive correlational relationship between quantity of milk added to cereal and the overall RAPID3 score decreased from 0.170 to 0.160 when only controlled for sex compared to when controlled for both age and sex. This suggested that in addition to sex, the age of the participants have an influence in controlling for the positive correlational relationship. In fact, of all the statistically significant correlational relationships that were examined, the highest correlational relationships were identified between the age of the participants and “difficulty walking two miles or more” (r=0.209) and “difficulty participating in recreational activities and sports” (r=0.202). Additionally, the prevalence of IA was greater in females and the results of the RAPID3 questionnaire seem to suggest that females experienced statistically significant higher overall self-reported disease activity of IA compared to males (Figure 1). This lends support to prior studies that indicated female patients with RA tend to have worse disease activity than their counterparts [27].

Despite being unable to identify statistically significant relationship between individual questions from the modified DHQ and the overall self-reported RAPID3 scores, 10 statistically significant correlational relationships were identified between individual questions from the modified DHQ and individual questions from the RAPID3 questionnaire (Table 2). A distinction must be made that these statistically significant relationships were identified in individual questions of the RAPID3 questionnaire, pertaining to the self-reported difficulties in performing individual activities of daily living, and not the overall self-reported disease activity of IA. Nevertheless, of these 10 statistically significant relationships, three were between the ages of the participants and questions from the RAPID3 questionnaire, while the remaining seven were between questions about dairy consumption and questions from the RAPID3 questionnaire.

**Table 2:** Significant values P-values of the two-tailed Pearson product-moment correlation was run between the individual variables of the modified DHQ and the individual variables of the RAPID3 questionnaire. DHQ - Diet history questionnaire RAPID3 - Routine Assessment of Patient Index Data 3

Sig. (2-tailed) Value Table															
	RAPID3 Questionnaire														
Diet History Questionnaire		1a.	1b.	1c.	1d.	1e.	1f.	1g.	1h.	1i.	1j.	2	3	RAPID 3 Score	
	Age	0.306	0.944	0.058	0.16	0.299	0.217	0.028	0	0.003	0.005	0.125	0.296	0.079	
	Q1.	0.259	0.313	0.178	0.827	0.48	0.256	0.477	0.073	0.46	0.223	0.95	0.989	0.752	
	Q1a.	0.305	0.301	0.102	0.279	0.098	0.024	0.21	0.107	0.256	0.132	0.43	0.473	0.312	
	Q2.	0.332	0.319	0.447	0.951	0.23	0.567	0.132	0.609	0.596	0.717	0.487	0.671	0.532	
	Q2d.	0.07	0.396	0.739	0.379	0.063	0.44	0.017	0.443	0.526	0.116	0.308	0.199	0.182	
	Q3.	0.689	0.812	0.682	0.406	0.24	0.834	0.486	0.246	0.227	0.886	0.258	0.964	0.811	
	Q3a.	0.224	0.681	0.375	0.575	0.825	0.19	0.883	0.877	0.674	0.501	0.546	0.697	0.981	
	Q4.	0.226	0.193	0.749	0.817	0.583	0.909	0.866	0.65	0.852	0.937	0.431	0.233	0.257	
	Q4a.	0.944	0.545	0.669	0.186	0.836	0.87	0.764	0.193	0.763	0.915	0.775	0.891	0.889	
	Q5.	0.956	0.651	0.266	0.364	0.842	0.733	0.885	0.353	0.338	0.36	0.433	0.7	0.692	
	Q5a.	0.809	0.426	0.1	0.41	0.155	0.899	0.841	0.821	0.973	0.435	0.812	0.242	0.592	
	Q7.	0.111	0.263	0.122	0.072	0.482	0.14	0.089	0.594	0.223	0.439	0.504	0.259	0.234	
	Q7a.	0.422	0.139	0.243	0.879	0.026	0.319	0.813	0.625	0.802	0.647	0.409	0.241	0.304	
	Q9.	0.929	0.167	0.156	0.105	0.566	0.165	0.733	0.267	0.643	0.29	0.602	0.602	0.459	
	Q9a.	0.357	0.094	0.875	0.218	0.943	0.271	0.78	0.989	0.522	0.384	0.937	0.436	0.77	
	Q10.	0.068	0.069	0.033	0.762	0.101	0.188	0.147	0.081	0.728	0.287	0.019	0.407	0.098	
	Q10a.	0.296	0.012	0.182	0.475	0.351	0.105	0.702	0.036	0.371	0.062	0.07	0.274	0.087	

Of these seven questions, debate remained whether the frequency of consumption or the quantity consumed with each consumption of dairy products had a greater impact on the self-reported difficulty of performing activities of daily living. In total, five statistically significant correlational relationships were identified between varying quantities of dairy products consumed, while only two statistically significant correlational relationships were identified between varying frequencies of consumption. Interestingly, all seven statistically significant correlational relationships were positive relationships, suggesting that an increase in either the quantity or the frequency of dairy products consumption led to increased difficulties with activities of daily living. However, in regard to quantity versus frequency, the quantity consumed with each consumption appeared to have a greater influence compared to the frequency of consumption in the past 12 months. If true, these results suggest that patients should prioritize consuming fewer quantities of dairy products with each consumption rather than consuming less frequently at the cost of consuming more each time.

One potential downside of this study is that only the correlational relationships between the self-reported disease activity of IA and frequency and quantity of dairy consumption was examined. Due to the design of the study, a cause-and-effect relationship is difficult to determine. However, for future studies, a prospective study or randomized control trial in which the patients are recruited and randomized into different groups with modifications to their diet with varying levels of dairy intake at different frequencies could be performed to assess if it makes an impact on the self-reported disease activity of IA. Furthermore, before guidelines for dairy consumption in IA patients can be developed, additional studies need to be conducted that control for the effect of pharmacologic agents.

Additionally, the validity of DHQ may be questioned in accurately recalling a diet from the past 12 months and the potential for recall bias. Nevertheless, multicenter randomized controlled trials have demonstrated the validity of DHQ to be strong particularly for frequency of intake of milk and dairy products in men and within acceptable range in women [28]. Similarly, questions on the validity of RAPID3 can be raised in accurately measuring the self-reported disease activity of RA and PsA. However, numerous studies have demonstrated that results from RAPID3 are comparable to other methods such as disease activity scored based on 28-joint count (DAS28), erythrocyte sedimentation rate (ESR), and clinical disease activity index (CDAI) [29,30].

Lastly, this study did not distinguish between participants who had RA versus those with PsA. However, recent studies suggest that there may be greater differences between the pathophysiology of RA and PsA, which may lead to differences in the effect of dairy on the self-reported disease activity [20,24].

## Conclusions

Overall, the results of this study suggest that there is no correlational relationship between dairy consumption and the overall self-reported disease activity of IA, given that the majority of the correlations identified in this study were non-significant and small in magnitude. However, whilst controlling for age and sex, two positive correlational relationships were identified between the quantity of milk consumed as a beverage (r=0.147) and the quantity of milk added to cereal (r=0.170) and the overall RlAPID3 score. No other questions from the DHQ demonstrated statistically significant correlational relationships with the overall RAPID3 score. Comparisons between the individual questions of the modified DHQ and the individual variables of the RAPID3 questionnaire resulted in seven positive correlational relationships being identified. Furthermore, variations in the quantity of dairy products consumed with each consumption appeared to have a greater impact compared to variations in the frequency of dairy consumed in the past 12 months. Despite these results, additional prospective studies would need to be performed before firm guidelines on dairy consumption and disease activity can be established. Nevertheless, results from this study suggest that avoidance of dairy products would not be a significant method for controlling the symptoms of IA.

## Figures and Tables

**Figure 3:**
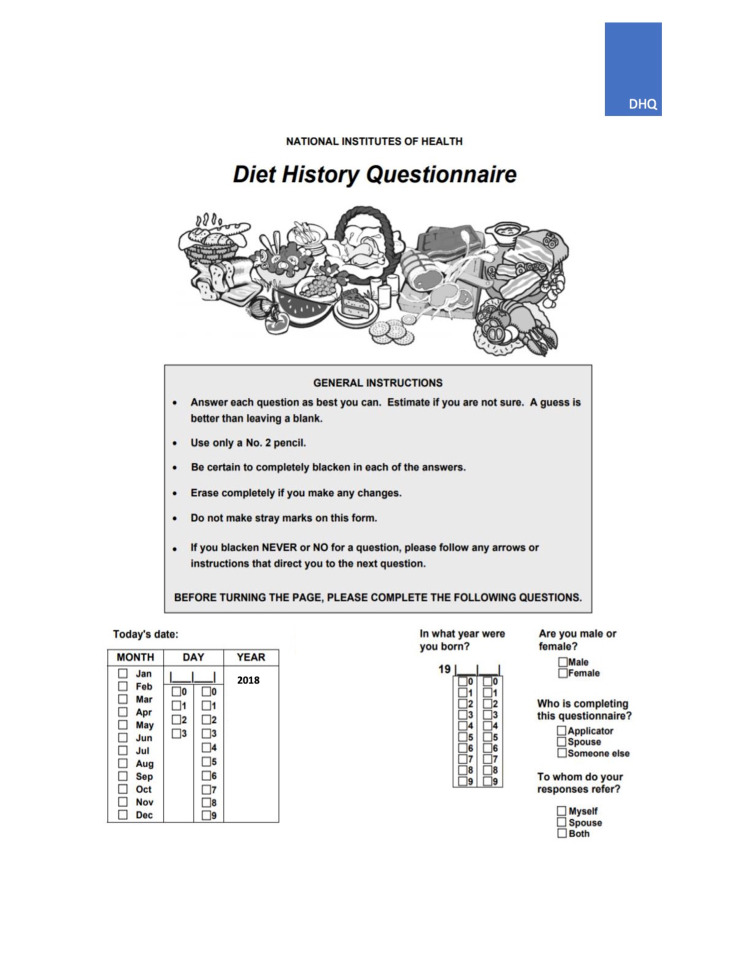
Modified diet history questionnaire: assessed patient's dairy intake within the last 12 months (page 1)

**Figure 4:**
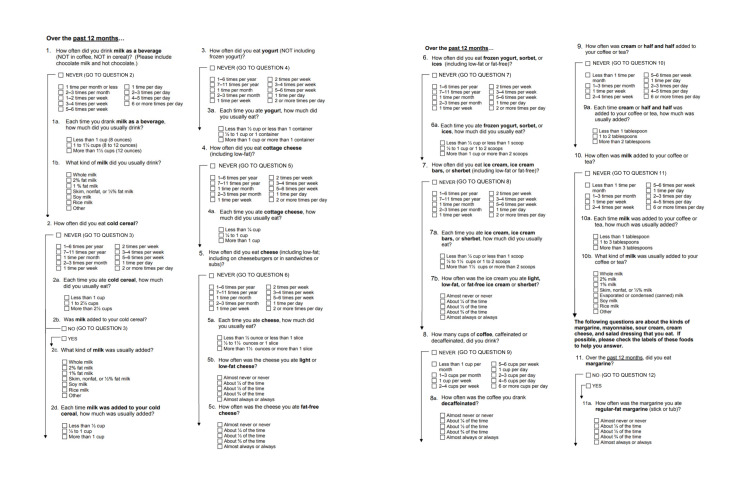
Modified diet history questionnaire (pages 2 and 3)

**Figure 5:**
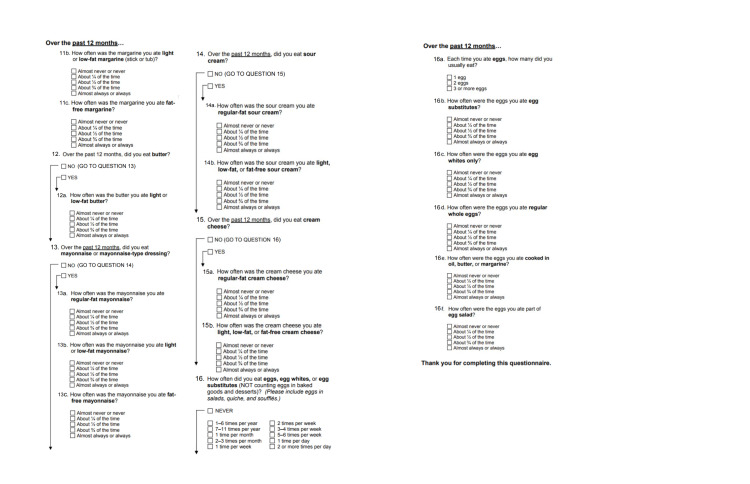
Modified diet history questionnaire (pages 4 and 5)

**Figure 6:**
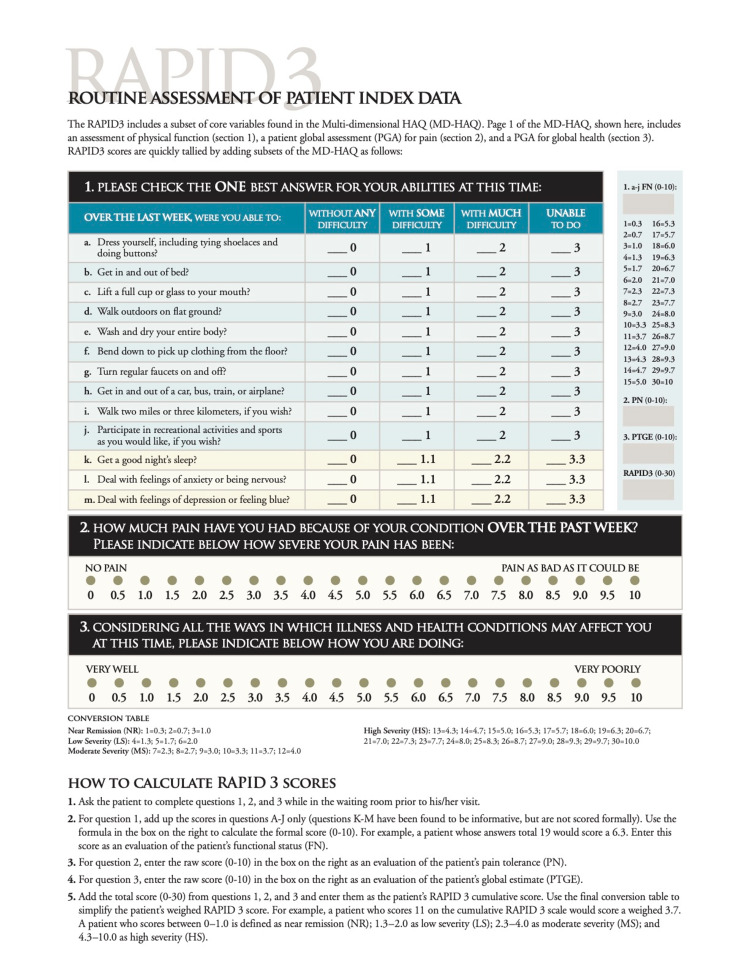
Routine assessment of patient index data 3 (RAPID3) questionnaire that measures patient's self-reported disease activity of inflammatory arthritis

## Appendices

Appendix I 

Appendix II 
